# Initial experience of a deep learning application for the differentiation of Kikuchi-Fujimoto’s disease from tuberculous lymphadenitis on neck CECT

**DOI:** 10.1038/s41598-022-18535-8

**Published:** 2022-08-19

**Authors:** Byung Hun Kim, Changhwan Lee, Ji Young Lee, Kyung Tae

**Affiliations:** 1 Department of Otolaryngology-Head and Neck Surgery, Hanyang University Hospital, College of Medicine, Hanyang University, 222 Wangsimni-ro, Seongdong-gu, Seoul, 04763, Republic of Korea; 2 Department of Biomedical Engineering, Hanyang University, Seoul, Korea; 3 Department of Radiology, Seoul St. Mary’s Hospital, College of Medicine, The Catholic University of Korea, 222 Banpodaero, Seocho-gu, Seoul, 06591 Republic of Korea

**Keywords:** Machine learning, Medical research, Autoimmune diseases, Tuberculosis, Classification and taxonomy

## Abstract

Neck contrast-enhanced CT (CECT) is a routine tool used to evaluate patients with cervical lymphadenopathy. This study aimed to evaluate the ability of convolutional neural networks (CNNs) to classify Kikuchi-Fujimoto’s disease (KD) and cervical tuberculous lymphadenitis (CTL) on neck CECT in patients with benign cervical lymphadenopathy. A retrospective analysis of consecutive patients with biopsy-confirmed KD and CTL in a single center, from January 2012 to June 2020 was performed. This study included 198 patients of whom 125 patients (mean age, 25.1 years ± 8.7, 31 men) had KD and 73 patients (mean age, 41.0 years ± 16.8, 34 men) had CTL. A neuroradiologist manually labelled the enlarged lymph nodes on the CECT images. Using these labels as the reference standard, a CNNs was developed to classify the findings as KD or CTL. The CT images were divided into training (70%), validation (10%), and test (20%) subsets. As a supervised augmentation method, the Cut&Remain method was applied to improve performance. The best area under the receiver operating characteristic curve for classifying KD from CTL for the test set was 0.91. This study shows that the differentiation of KD from CTL on neck CECT using a CNNs is feasible with high diagnostic performance.

## Introduction

In benign cervical lymphadenopathy, both Kikuchi-Fujimoto disease (KD) and cervical tuberculous lymphadenitis (CTL) can show enlarged cervical lymph nodes with or without necrosis, with similar imaging features on contrast-enhanced CT (CECT)^1–5^. Although both are benign, there are differences in their treatment and course^6–11^. Therefore, many studies have been performed to differentiate KD from CTL using CECT^1–3,12^. KD can show perinodal infiltration, indistinct margins of necrosis, stronger cortical enhancement of lymph nodes, and unilaterality^1,2,12,13^. CTLs can demonstrate a lower density of necrosis, unilocular necrosis, calcifications, and skin fistula on CECT^4,9^. However, the differential diagnosis using imaging alone is not easy. The condition needs to be confirmed by histopathologic diagnosis using fine needle aspiration, core needle biopsy, or excision^13–16^. However, because these histopathologic confirmations are invasive, additional information from convolutional neural networks (CNNs) could be useful to enhance conspicuity for deciding the diagnosis.

Deep learning methods with CNNs utilize multiple layered neural networks to develop robust predictive models without feature selection by human image evaluation experts^17–20^. In radiology, many studies using CNNs have focused on the detection or classification of lesions and the validation of the deep learning technique performance^20–35^. The performance of CNNs has been improved and found to be comparable to that of radiologists in many studies^32–36^. However, the deep learning application to neck CT could have many challenges in the detection and classification of cervical lymphadenopathy, because neck CT has many anatomic structures from skull base to thoracic inlet and CT has inferior soft tissue contrast to MR^37–41^. Accordingly, for the enhancement of performance, this study used the Cut&Remain method^40^, which is one of supervised augmentation methods and crops the image to focus on the lesion.

Recently, in head and neck imaging, deep learning methods have been used to differentiate metastatic lymph nodes in thyroid cancer, discriminate benign and malignant thyroid nodules, detect extracapsular extension of metastatic lymph nodes in head and neck cancers, and automatic lymph nodes segmentation^20,24,34–36,41^. However, no studies have investigated the feasibility of deep learning applications to classify of benign cervical lymph nodes. In this study, a deep learning method was developed to discriminate KD from CTL on CECT. The purpose of this study was to evaluate the ability of CNNs to differentiate benign cervical lymphadenopathy and classify KD and CTL in patients with benign cervical lymphadenopathies.

## Results

### Patient characteristics

Among the study cohort, KD occurred more frequently in women (75.2%) and the patients’ age of KD was significantly younger (mean age, 25.1 ± 8.7 years) than CTL (mean age, 41.0 ± 16.8 years) (p = 0.002 and < 0.001, respectively). The most common symptom was a palpable cervical mass, which was observed in 99.2% of patients with KD (124 of 125 patients) and 97.3% of patients with CTL (71 of 73 patients). Fever was more frequently observed in patients with KD (60.0%, 75 of 125 patients) than in those with CTL (5.5%, 4 of 73 patients). There was no significant difference in cervical lymph node enlargement unilaterality (p = 0.070) [95.2% for KD (119 of 125 patients) and 74.0% for CTL (54 of 73 patients)]. The abbreviations of the study are listed in Table 1. The detailed data of the study population are summarized in Table 2.

**Table 1:** List of abbreviations.

Abbreviation	Meaning
AUC	Area under the receiver operating characteristic curve
CAM	Class activation map
CECT	Contrast-enhanced computed tomography
CNNs	Convolutional neural networks
CTL	Cervical tuberculous lymphadenitis
Eq	Equation
Grad-CAM	Gradient-weighted class activation map
KD	Kikuchi-Fujimoto's disease
LN	Lymph node
ROC	Receiver operating characteristic

**Table 2:** Demographics and clinical characteristics of study patients with Kikuchi-Fujimoto disease (n = 125) and cervical tuberculous lymphadenitis (n = 73).

Diagnosis	KD (n = 125)	CTL (n = 73)	P value
**Sex**			0.002*
Men	31 (24.8)	34 (46.6)	
Women	94 (75.2)	39 (53.4)	
Ages (years)	25.1 ± 8.7	41.0 ± 16.8	< 0.001*
Men (mean ± standard deviation)	21.5 ± 8.0	39.4 ± 15.1	< 0.001*
Women (mean ± standard) deviation)	26.3 ± 8.6	42.3 ± 18.2	< 0.001*
**Symptoms**			
Neck mass	124 (99.2)	71 (97.3)	0.354
Fever	75 (60.0)	4 (5.5)	< 0.001*
Headache	17 (13.6)	1 (1.4)	< 0.001*
Myalgia	8 (6.4)	1 (1.4)	0.054
Weight loss	3 (2.4)	4 (5.5)	0.309
**Sites of lymphadenopathy**			0.070
Unilateral	119 (95.2)	54 (74.0)	
Bilateral	6 (4.8)	19 (26.0)	
**Pathologic diagnosis**			
Fine needle aspiration	16 (12.8)	18 (24.7)	
Core needle biopsy	89 (71.2)	23 (31.5)	
Excision	20 (16.0)	32 (43.8)	

*p < 0.05.

### Diagnostic performance for classification

**Figure 1:**
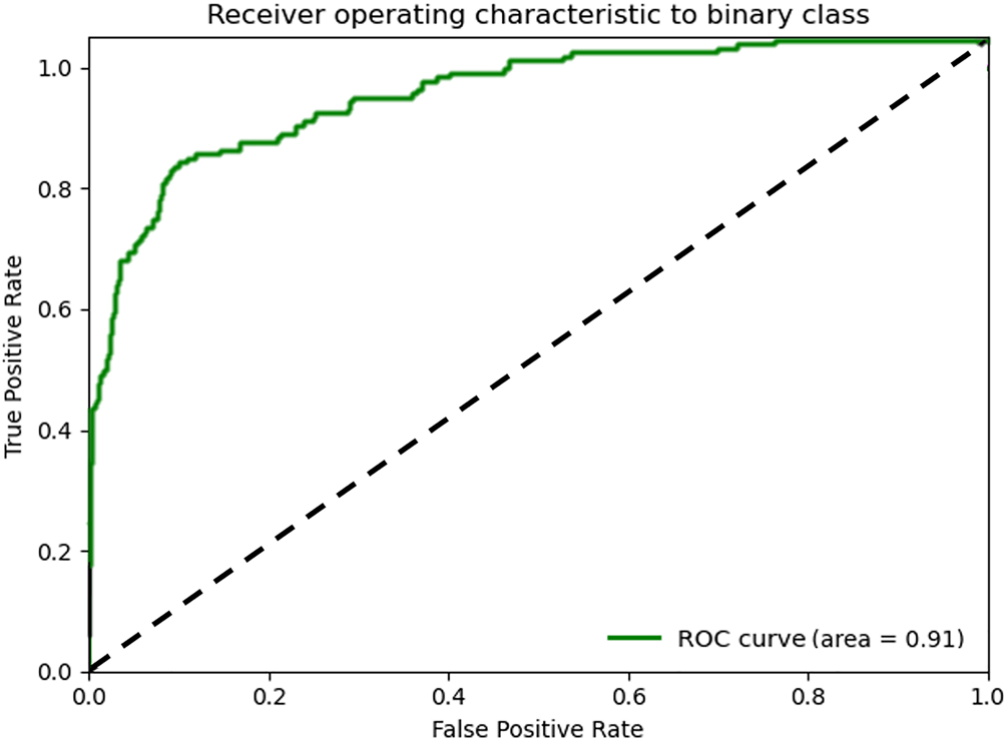
The ROC curve of CNNs for the differentiation of Kikuchi-Fujimoto disease from cervical tuberculous lymphadenitis. The CNNs with application of Cut&Remain technique (aspect ratio = 3.0) shows an AUC of 0.91.

The diagnostic performance of the deep learning models is shown in Table 3. In the preprocessing, the CT images were divided into three groups, as follows: the original image with aspect ratios of 1.0, 1.5, and 2.0, the original image with an aspect ratio of 3.0, and the original image with an aspect ratio of 4.0. The test results of each group showed accuracies of 69.15%, 94.67%, and 86.05%, respectively. The aspect ratio of 3.0 setting showed an accuracy of 94.67%, a sensitivity of 99.52%, a specificity of 73.90%, a positive predictive value of 94.22%, and a negative predictive value of 97.32%, representing the best diagnostic performance. For the test set (1266 slices), the area under the receiver operating characteristic curve (AUC) of CNNs was 0.91 with an aspect ratio of 3.0, followed by 0.87 with an aspect ratio of 4.0 (Fig. 1).

**Table 3:** Diagnostic performance for classification.

ResNet-50	Original image with aspect ratios = {1.0, 1.5, 2.0}	Aspect ratio = {3.0}	Aspect ratio = {4.0}
Accuracy (%)	69.15	94.67	86.05
Sensitivity (%)	71.93	99.52	88.98
Specificity (%)	57.29	73.90	73.56
PPV (%)	87.80	94.22	93.50
NPV (%)	32.31	97.32	60.96
AUC	0.71	0.91	0.87
F1-score	0.74	0.97	0.91

*PPV* positive predictive value, *NPV* negative predictive value, *AUC* area under the receiver operating characteristic curve.

### Qualitative evaluation by Grad-CAM

**Figure 2:**
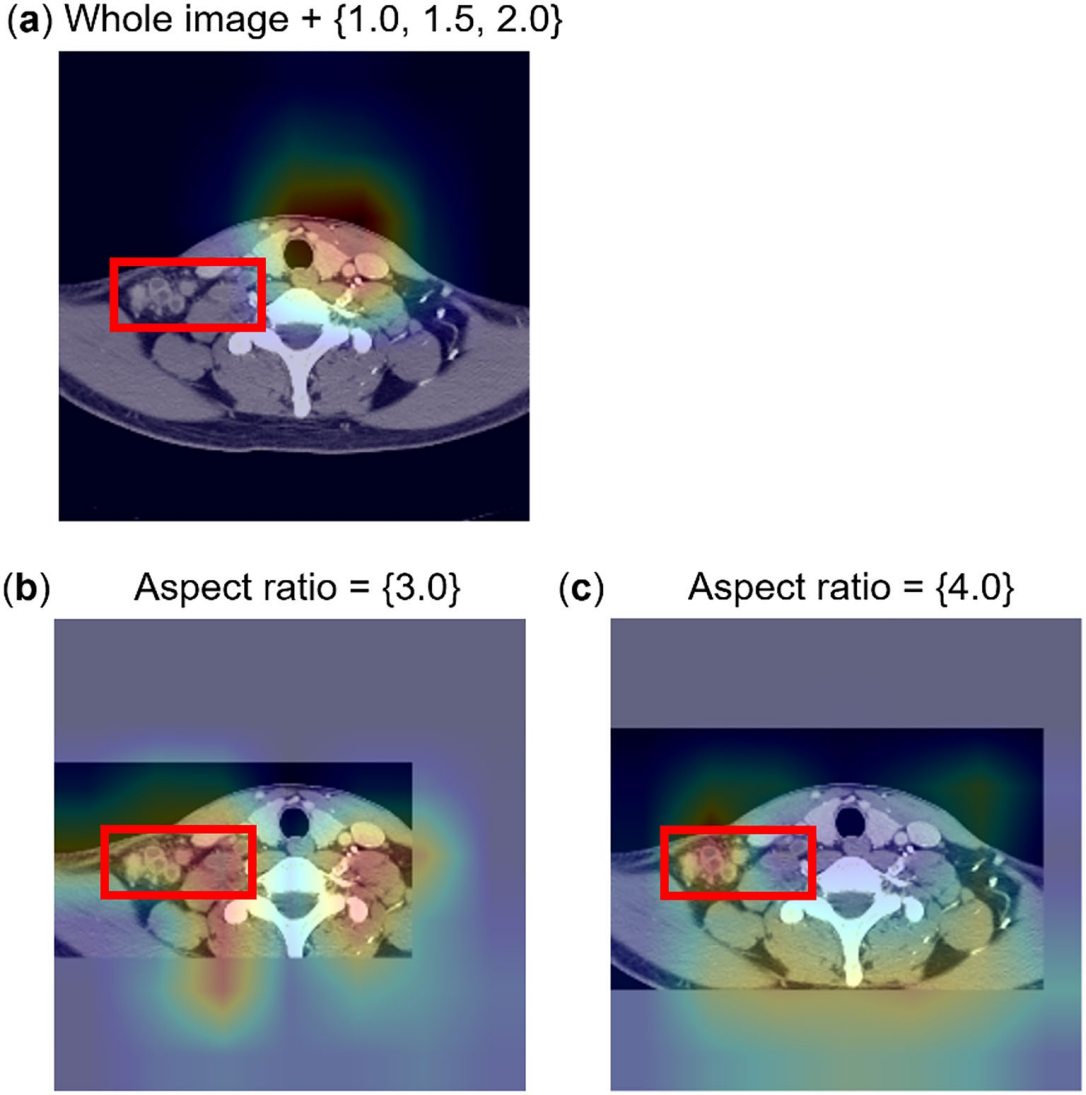
Representative attention guide with CAM images in each Kikuchi-Fujimoto disease and cervical tuberculous lymphadenitis group. This figure shows test examples as well as the corresponding Grad-CAM according to the aspect ratio. Ground-truth annotation are shown with a red box. In case of aspect ratios (**b**) 3.0 and (**c**) 4.0, the Grad-CAM results indicate that the trained model identify the enlarged lymph nodes in right level IV and supraclavicular fossa.

An augmentation technique was developed, where labeled lesions were mainly considered as cues for classification, with attention-guided networks^39^. To verify that the technique was indeed learning to recognize the lesions in target images, the activation maps for test images were visually shown. The vanilla ResNet-50 model was used to obtain Grad-CAM to observe the effect of the augmentation method clearly. Figure 2 shows the test examples and the corresponding Grad-CAM according to the aspect ratio. For aspect ratios of 3.0 and 4.0, Grad-CAM indicated enlarged lymph nodes in the right level IV and supraclavicular fossa. Grad-CAM analysis indicated that Cut&Remain force a model to focus on lesions, irrespective of the background.

## Discussion

This study developed a deep learning algorithm for classifying KD and CTL in patients with benign cervical lymphadenopathy using neck CECT images. With the application of a bounding box, the deep learning algorithm exhibited remarkable performance in classifying KD and CTL. The results indicated that a CNNs could accurately distinguish KD from CTL with an AUC of 0.91 in the test dataset. Therefore, we expect the application of CNNs to be feasible for classifying cervical lymphadenopathy.

Recently, deep learning techniques have been validated for head and neck oncology imaging^26,34–36^. A prior study applied a deep learning method to diagnose metastatic lymph nodes and identify extracapsular extension in head and neck cancer, with an AUC of 0.91. Another study showed the high performance of deep learning CNNs, with an AUC of 0.95 for diagnosing metastatic lymph nodes in patients with thyroid cancer on CECT. This study result also showed a high performance, with an AUC of 0.91 for classifying KD and CTL on CECT. This study result was enhanced when the bounding boxes were applied using the Cut&Remain method. We hypothesize that dedicated comparisons between KD and CTL will be possible when expert experience and supervision are added.

Among benign cervical lymphadenopathies, KD and CTL are the major differential diagnoses in patients with acute cervical lymphadenopathy^3^. In prior studies with CT, the presence of indistinct margins of necrotic foci was found to be an independent predictor of KD, with 80% accuracy^1^. Another previous study demonstrated that bilateral involvement, ≥ five levels of nodal involvement, absence or minimal nodal necrosis, marked perinodal infiltration, absence of upper lung lesion, and mediastinal lymphadenopathy were independent findings that suggest KD rather than tuberculosis on CT. The investigators reported an AUC of 0.761 for these five CT findings, which was considerably lower than this result^2^. We suggest that combined CT findings and simultaneous deep learning could enhance diagnostic performance for benign cervical lymphadenopathy.

There have been many studies on cervical lymph node analysis using deep learning, particularly in oncology^34,36,39^. However, no previous investigations have evaluated the application of a deep-learning model to discriminate benign cervical lymphadenopathy. Notably, Adele discriminated between normal lymph nodes and lymphadenopathy; however, detailed lymph node disease classification was not covered^41^. In this study, the CNNs algorithm has several advantages. First, it shows higher performance than previous qualitative analysis studies. The performance could be related to the application of the Cut&Remain method, which is a simple and efficient supervised augmentation method. This method drives a model to focus on relevant subtle and small regions; therefore, it is possible to differentiate such small lesions from medical images and enhance performance. This method has been used to classify clavicle and femur fractures on radiography and 14 lesion classifications on chest radiographs. Previous studies have shown improved performance with the application of Cut&Remain^40^; this study demonstrated the best performance with the Cut&Remain method as well. We believe that as there are many anatomic structures on neck CECT, this method could be effective in future studies using deep learning applications for neck CT imaging.

This study had several limitations. First, the subject number of CTL was smaller than that of KD. Accordingly, to overcome this problem, data augmentation was performed on CTL. Second, it is important to differentiate malignant from benign lymph nodes, and this study aimed to differentiate and classify benign cervical lymph nodes only. Further studies are needed to distinguish metastatic from benign lymph nodes. Third, an external validation was not performed, and the deep learning algorithm may exhibit overfitting results. Finally, we did not evaluate radiologist performance and compared radiologist performance with that of the deep learning method. A study that investigates this will be further conducted in the future.

In conclusion, the deep learning method is helpful in the differentiation of benign cervical lymphadenopathy between KD and CTL. The performance of CNNs method can be enhanced with the Cut&Remain method. In the future, deep learning diagnostic algorithm could be developed for differentiation of cervical lymphadenopathy including benign and malignant lymph nodes with large number of patients.

## Methods

This study was approved by the institutional review board (IRB) of Hanyang University Seoul Hospital (2020-07-048-004). The requirement of informed consent was waived due to its retrospective nature by the institutional review board (IRB) of Hanyang University Seoul Hospital. All experiments were performed in accordance with relevant guidelines and regulations.

### Patients and datasets

**Figure 3:**
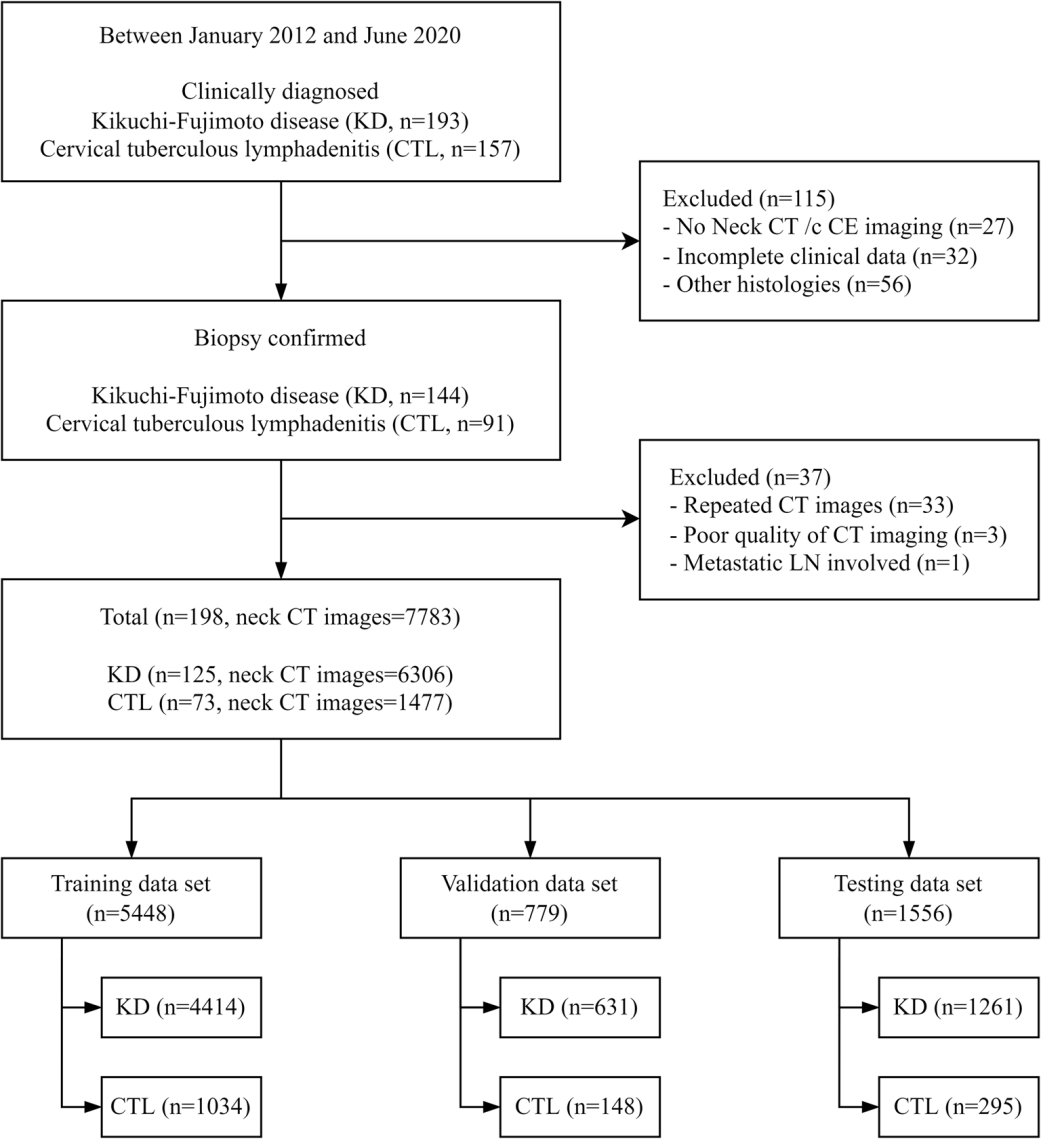
Flow chart of the study population.

We retrospectively investigated the medical records of 350 patients who were clinically diagnosed with KD and CTL from January 2012 to June 2020 at a tertiary hospital (193 KD, 157 CTL). Patients were excluded if they did not undergo pathologic confirmation (n = 56), lacked a neck CECT (n = 27), had incomplete clinical data (n = 32), or had a poor quality CECT due to metal dental artifacts (n = 3). Finally, 125 patients with KD and 73 with CTL were included. A flowchart of patient selection is shown in Fig. 3. All scans of neck CECT were downloaded from PACS in the DICOM format. A neuroradiologist and an otolaryngology resident reviewed the images and CT scans demonstrating only enlarged cervical lymph nodes were included. Finally, patients with KD consisted of 6306 slices, and those for CTL consisted of 1477 slices. The training datasets comprised training (4414 slices for KD and 1034 slices for CTL) and validation (631 slices for KD and 148 slices for CTL) slices. The test datasets were composed of 1261 slices for the KD group and 295 for the CTL group.

### CT imaging protocol

Contrast-enhanced CT imaging was performed after the administration of intravenous iodine contrast agent (1.2 mL/kg, 2 mL/s, 30 s delay) using 120 kVp, 200 mAs, and 2 mm slice thickness reconstruction (Brilliance 64, Philips Healthcare, Best, The Netherlands; SOMATOM, Definition Flash, Siemens Healthcare, Erlangen, Germany).

### Labeling

**Figure 4:**
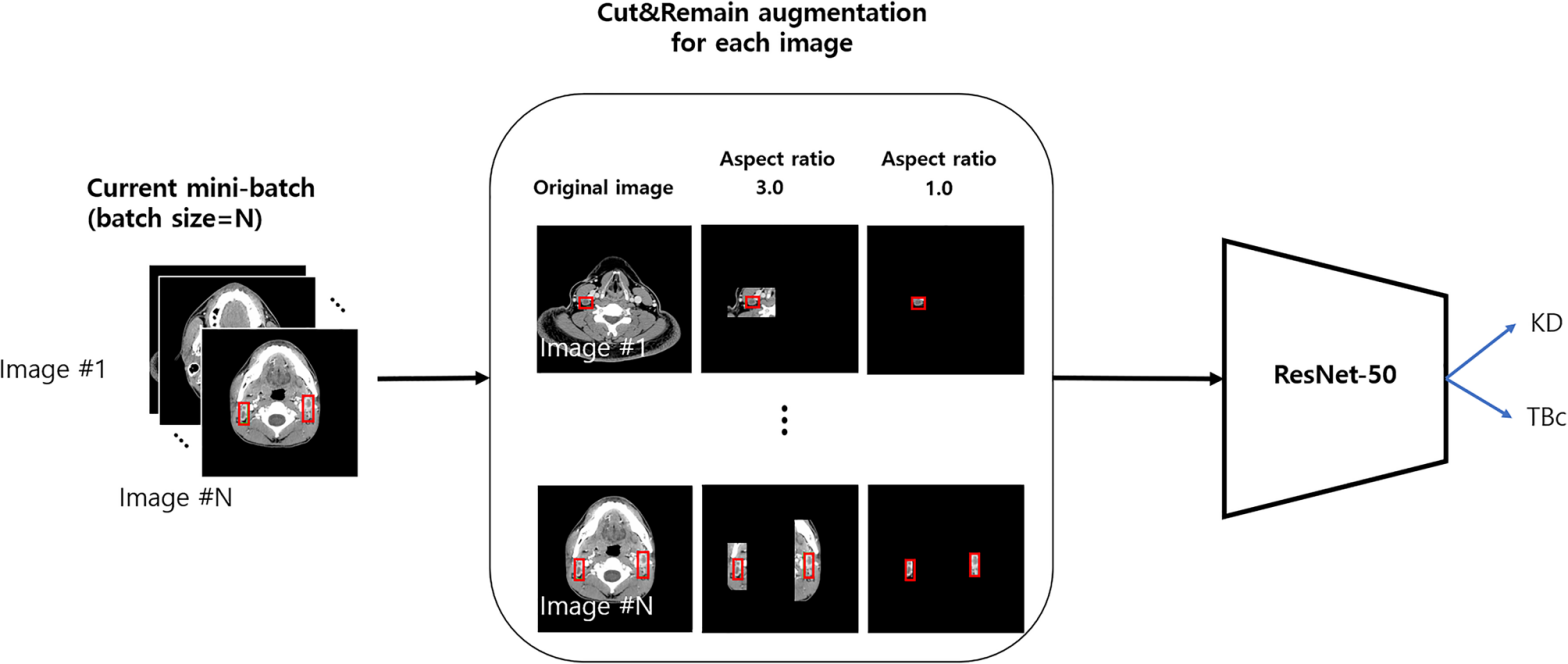
Pipeline of the CNNs for the differentiation of Kikuchi-Fujimoto disease from cervical tuberculous lymphadenitis.

Figure 4 shows a schematic representation of the pipeline for the differentiation of KD from CTL. A radiologist (J.Y.L, a neuroradiologist with nine years of experience) manually identified cervical lymph node lesions on the CT images and then drew a rectangular bounding box on the cervical lymph nodes. The image was cropped based on the bounding box-labeled image. The labeled image was used as an augmentation technique to generate a new training sample.

### Data augmentation technique to identify local key features

A novel data augmentation technique, called Cut&Remain, was applied to eliminate unimportant portions of the image while preserving the spatial location of the important areas^40^.

**Figure 5:**
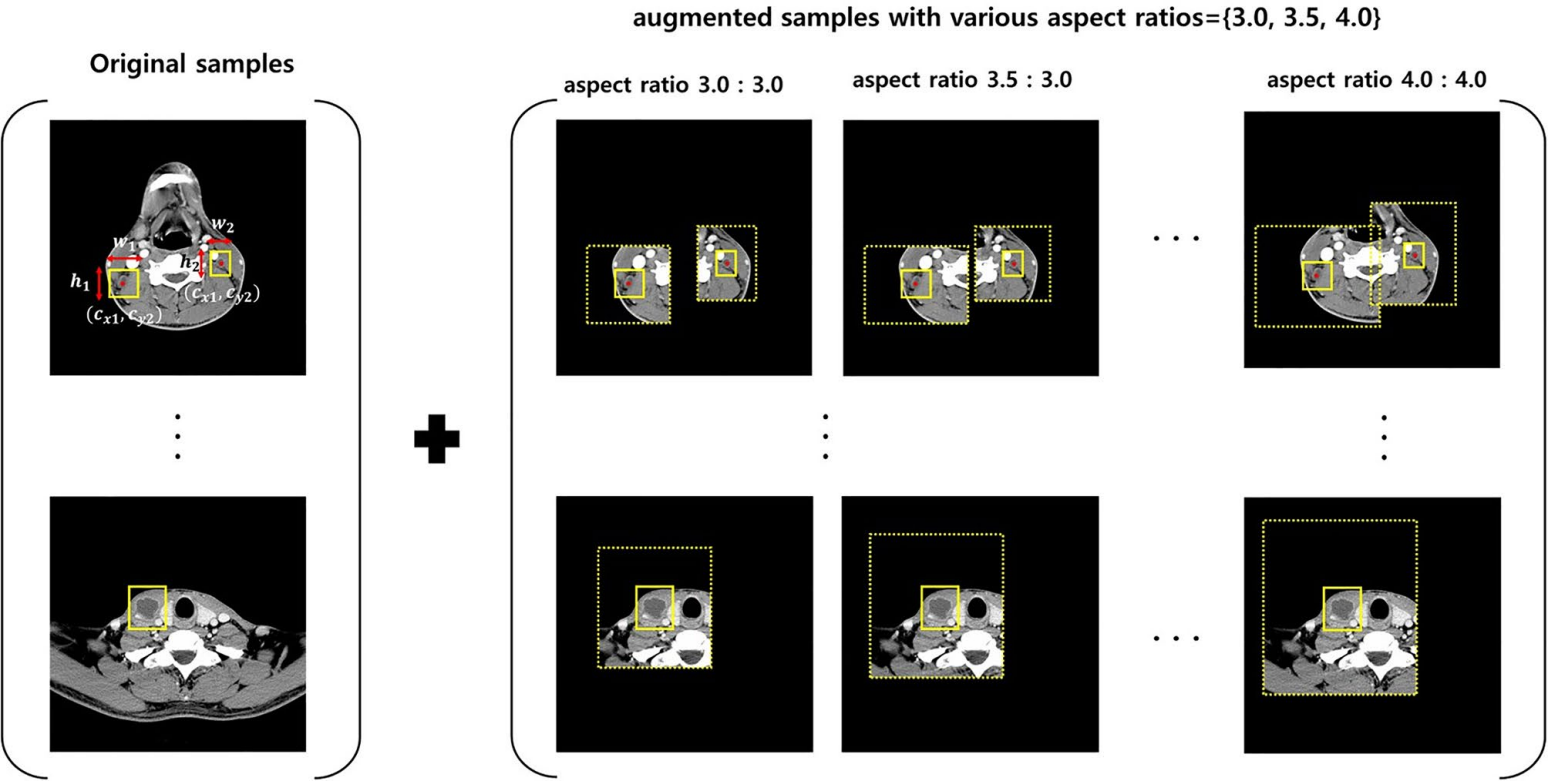
Mini-batch configuration using Cut&Remain data augmentation during training.

The CNNs assumed that $x\in {\mathbb{R}}^{W\times H\times C}$ and $y$ denote the training image and label, respectively. The goal of Cut&Remain technique is to generate a new training sample $(\widetilde{x},\widetilde{y})$, which can be described by Eq. (1) and used to train the model based on its original loss function.

1 $$\begin{gathered} \tilde{x} = M \odot x, \hfill \\ \tilde{y} = y \hfill \\ \end{gathered}$$

where $\mathrm{M}\in {\{\mathrm{0,1}\}}^{W\times H}$ denotes a binary mask indicating lesion and $\odot$ is element-wise multiplication. To generate mask, $M$, a bounding box annotation $\mathrm{B}=({c}_{x},{c}_{x},\mathrm{ w},\mathrm{ h})$, was used to indicate the cropped region on image $x$. For the bounding boxes, nine aspect ratios of {2.0, 2.5, 3.0} were considered. Within the cropped region, the value of binary mask $\mathrm{M}\in {\{\mathrm{0,1}\}}^{W\times H}$ was 1 within B or 0 outside B. In each training step, an augmented sample $(\widetilde{x},\widetilde{y})$ was generated based on each training sample according to Eq. (1) and included in the mini-batch, as shown in Fig. 5.

Data augmentation was performed with rotation (− 10° to 10°), flip (horizontal 50%), zoom (95–105%), and translation shift (0–10% of the image size in the horizontal and vertical axes), which was applied before Cut&Remain augmentation with corresponding bounding boxes.

### Deep learning training strategy

ResNet-50 was adapted as a backbone network and we have initialized the weights randomly. We used binary cross-entropy loss for classification and Adam with a momentum of 0.9. The initial learning rate was set to 0.0005. The model was trained for 2000 epochs in total, and the learning rate was reduced by a factor of 10 at 1000 epochs.

### Statistical analysis

The sensitivity, specificity, and AUC were evaluated to assess the diagnostic performance of the deep learning algorithm. We report the AUC and average F1-score of five random runs with different initializations for the classification performance.

## Data Availability

The datasets generated for the current study are available from the corresponding author on reasonable request.

## Code availability

The codes generated for the current study are available from the corresponding author on reasonable request.
